# Analysing mathematical modelling tasks in light of citizenship education using the COVID-19 pandemic as a case study

**DOI:** 10.1007/s11858-022-01440-9

**Published:** 2022-10-19

**Authors:** Katja Maass, Stefan Zehetmeier, Anika Weihberger, Katharina Flößer

**Affiliations:** 1 International Centre for STEM Education (ICSE), University of Education Freiburg, Freiburg, Kunzenweg 21, 79117 Freiburg, Germany; 2 Institute of Instructional and School Development, University of Klagenfurt, Sterneckstraße 15, 9020 Klagenfurt, Austria

**Keywords:** Mathematical modelling, Authentic contexts, Numeracy, Socio-scientific issues, Citizenship education

## Abstract

In this paper, we discuss the theoretical background of mathematical modelling and its connection to citizenship education. Citizenship education in this context means that young people are equipped with competencies to respond as responsible citizens in situations relevant for society. To outline the connection between mathematical modelling and citizenship education in theory, we discuss the aims of mathematical modelling, modelling competences and the connection between numeracy and modelling. Based on these reflections we present an extended modelling cycle that specifically highlights modelling steps relevant to citizenship education. To show how the theoretical connection between mathematical modelling and citizenship education can be used in teaching practice, we describe three different examples of modelling tasks and analyse them with the help of the extended modelling cycle. We argue that the three tasks support different learning aims in relation to citizenship education and require modellers to carry out different steps of the extended modelling cycle. As an example of context, we used the pandemic caused by COVID-19, as it affected the quality of human life greatly, as all students in the Western world experienced.

## Introduction

In this paper, we discuss the theoretical background of mathematical modelling and its connection to citizenship education. Citizenship education in this context means that young people are equipped with competences to respond as responsible citizens in situations relevant for society. Learning how mathematical modelling leads to prognosis and decisions is an essential step to realising citizenship education because it helps citizens learn about mathematics and how it interacts with reality, leading to better acceptance of science (Weisberg et al., 2021).

To connect mathematical modelling and citizenship education in theory, we first discuss how the aims of modelling relate to responsible citizens’ education. Second, we need to know which competences students need to reach the aims of modelling, as discussed. To clarify this point, we discuss the definition of modelling competences. Based on this definition, we extend our understanding of modelling competences and the modelling cycle, by connecting numeracy and modelling.

Within the many different perspectives of modelling, the social-critical perspective emphasises a critical understanding of the surrounding world as a central aim of modelling (Kaiser & Sriraman, 2006). Drawing on this perspective, we define modelling competences by means of a list of sub-competences (“bottom-up approach”, Cevikbas et al., 2021; Niss & Blum, 2020) and use insights from numeracy (Geiger et al., 2015a, 2015b) and socio-scientific issues (controversial issues relevant for society that require decision-making,. Ratcliff & Grace, 2003) to develop a clear vision about how modelling connects with the aim of citizenship education. Based on these theoretical reflections, we present an extended modelling cycle (Maass et al., 2019) and consequently an extended list of modelling sub-competences that specifically highlight modelling steps relevant to the aim of citizenship education. This extended modelling cycle can be used to analyse the didactical potential of modelling tasks in relation to citizenship education. It allows us to show which aspects of a modelling task can give insight into processes that are carried out when, for example, political decisions are made on controversial issues relevant for society.

To show how the theoretical connection between modelling and citizenship education can be used in teaching practice, we developed three generic examples of modelling tasks and analysed them didactically. We argue that the three tasks support different learning aims related to citizenship education and require modellers to carry out different steps of the extended modelling cycle.

While other contexts relevant for society such as climate change can be used for developing modelling tasks, we chose COVID-19 because it has recently led to challenging times for human society. COVID-19 has caused millions of deaths, directly affected many people through illness and led to substantial restrictions in our lives. This pandemic has revealed the need for all citizens to understand and appreciate the role of scientific proceedings and arguments, particularly mathematical modelling (Aguilar & Castaneda, 2021).

## Theoretical background

Since this paper’s goal is to discuss mathematical modelling and its connection to citizenship education, we therefore firstly look at the aims of modelling to see if it makes sense to connect modelling with the overall aim of citizenship education (Sect. 2.1).

Once this goal is clarified, we look at the competences needed to reach these aims. By doing so, we follow the approach of Kaiser and Sriraman (2006), who link different perspectives on modelling with competences and skills. For this, we need to outline our understanding of modelling competences based on sub-competences related to the modelling cycle (Sect. 2.2).

We extend this understanding of modelling competences (in Sect. 2.3) by connecting mathematical modelling with numeracy and socio-scientific issues, thus following up on the aim of citizenship education and the aims of modelling. This leads to an extended modelling cycle (Sect. 2.4), and thereby to a new set of modelling sub-competences connected with the aim of citizenship education.

### Aims of mathematical modelling

To see whether modelling can be connected to the overall aim of citizenship education, we take a detailed look at the aims connected with modelling.

There exist different theoretical perspectives on the aims of modelling. The so-called *integrative view* links a comprehensive set of objectives to include modelling in teaching (Blum, 1996; Kaiser, 1995). Based on this integrative perspective, Maass (2004) distinguished the following aims of modelling:

- *Methodological aims*: these entail developing students’ competencies in applying mathematics in simple and complex real-world situations.
- *Culture-related aims*: these give comprehensive insight into mathematics as a science and its value for our culture and society.
- *Pragmatic aims*: these involve understanding real-world situations and dealing with them.
- *Psychological aims*: these entail developing positive attitudes towards mathematics and supporting the memorizing and understanding of mathematical content.
- *Pedagogical aims*: these have the aim of developing competences in problem-solving, in reasoning, and in being creative.

The integrative perspective on modelling developed over time as did other perspectives. In their comprehensive analysis of different modelling perspectives, Kaiser and Sriraman (2006) distinguished, amongst others, the following perspectives: the realistic perspective, which emphasises the ability to solve real-world problems, understanding of the world, and the development of modelling competences; the educational perspective, which is related to the above-described integrative perspective and aims at structuring mathematical learning processes and learning mathematical concepts; and the social–critical perspective, which emphasises the critical understanding of the surrounding world (Kaiser & Sriraman, 2006). The importance of this classification is shown in its elaboration by Abassian et al. (2020).

In relation to the goal of connecting modelling to citizenship education, therefore, we update our aims for students’ learning as follows:

1. Learning to apply mathematics to their everyday and professional lives (former methodological aims, Maass, 2004; related to solving real-world problems in the realistic perspective);
2. Developing modelling competences (similar to methodological aims, realistic perspective);
3. Learning to understand their world critically (focus of socio-critical modelling, also developed further from the pragmatic aims);
4. Gaining insight into the usefulness of mathematics (culture-related aim);
5. Understanding and memorise mathematical content more easily and developing a more positive attitude towards mathematics (psychological aims);
6. Learning problem-solving and reasoning (pedagogical aims).

This discussion indicates that it is indeed meaningful to connect modelling to citizenship education. Particularly, the aims A1–A4 emphasise the connection between modelling and reality. Therefore, they are particularly relevant for connecting modelling and the overall aim of citizenship education.

What tangible modelling competences do students need to develop to reach these aims? In 2.3 we provide our understanding of modelling and modelling competences and reflect on how this extended understanding is still in line with these aims of modelling. Before we can do so, we need to outline our definition of modelling competences.

### Modelling competences

In this section, we elaborate on modelling competences. Based on this understanding, we extend our definition of modelling competences and the modelling cycle in relation to the aims as outlined in Sect. 2.1.

Niss and Blum (2020) discussed two different perspectives on the notion of modelling competence—"top-down” and “bottom-up” (pp. 79–80). In the “top-down” perspective, holistic modelling competence is the primary objective, and its major components (modelling sub-competences or competences) are essential but subservient to the primary objective. The “bottom-up” view deals with a set of distinct and separate modelling sub-competences tightly linked to the modelling cycle without, in the first place, identifying them as components of an overarching modelling competence. Cevikbas et al. (2021) used these perspectives, among others, to analyse existing research on modelling, and found out that 75% of the reviewed research used the bottom-up perspective of modelling.

Obviously, following the “bottom-up” view does not capture the whole picture, as there are many aspects of modelling competences which are not linked to the individual steps of the modelling cycle but are overarching. Relevant overarching competences identified by research, for example, are as follows:

- metacognition (Stillman, 2011; Schukajlow et al., 2021; Vorhölter, 2018, 2021);
- creativity (Lu & Kaiser, 2022);
- reading competences (Krawitz et al., 2022).

Still, in this paper, we follow the bottom-up approach which deals with a set of distinct and separate modelling sub-competences tightly linked to the modelling cycle. This perspective allows us to extend the modelling cycle and therewith our understanding of modelling competences, in order to target the aims connected to modelling as discussed in Sect. 2.1.

By doing so, we do not claim that metacognition or creativity are not important. Maass (2004) also followed the bottom-up approach and defined modelling competences according to the sub-competences carrying out the single steps, but also including metacognition and competences in reasoning. Here, however, our focus is on the sub-competences connected to the modelling cycle.

**Fig. 1:**
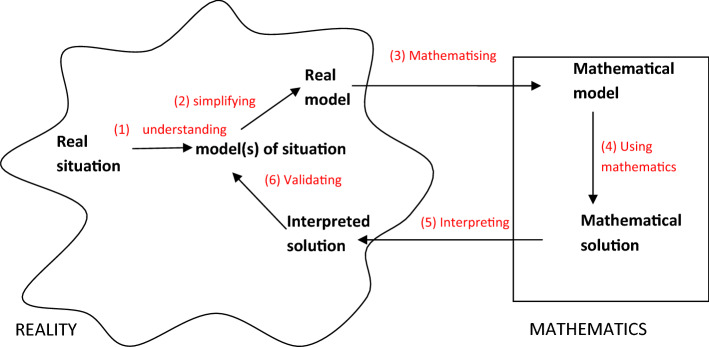
Modelling cycle according to Blum and Leiss (2007)

When one follows the bottom-up approach tightly linked to a modelling cycle, the definition of the modelling cycle on which one bases it is important. Here, we illustrate the definition of modelling competences based on the commonly known conceptualisation by Blum and Leiss (2007) (Fig. 1).

Thus, in this case, the modelling competences comprise six sub-competencies, ranging from (1) setting up a situation model by understanding the situation to (6) validating the solution (Fig. 1). As acknowledged above, modelling competences can include further competences such as metacognition or creativity. To analyse our three tasks, we focus just on the sub-competences. Surely, the sub-competences are closely connected to reaching the overall aim of citizenship education. But as our discussion in Sect. 2.3 shows, there are further sub-competences (and related steps in the modelling cycle) which are not included in this list and which can be included to emphasize the aim of citizenship education. We come back to this definition of modelling competences in 2.3.

To acquire modelling competences, it is important to include tasks that require following the whole modelling cycle (*holistic tasks*) and tasks in which only single steps of the modelling cycle need to be performed (*atomistic tasks*) (Blomhoej & Jensen, 2003). Maass and Mischo (2011) developed such a modelling course for low-achieving students by combining tasks that require students to perform the whole modelling cycle and other tasks based on individual modelling steps. They showed that low-achieving students can acquire modelling competences when the teacher follows this modelling course (Mischo & Maass, 2013).

### Numeracy, modelling and citizenship education

In this section, we extend our understanding of modelling competences by connecting mathematical modelling with numeracy and socio-scientific issues.

The pandemic has revealed the need for all citizens to understand modelling cycles and judge situations based on the results of modelling cycles. People need to understand that scientific facts, as well as ethical, economic, political and social reasoning, influence—for example—decisions about restrictive measures that should be taken. Decision-makers need to make a cost–benefit analysis when considering how to contain the virus and the implications for the mental health of the population. Therefore, to learn to act as responsible citizens, students need to learn how to make decisions based on modelling results.

As outlined in Sect. 2.1, the aims for modelling include developing a critical worldview, which is particularly emphasised in the socio-critical perspective. Barbosa (2006), an advocate of this perspective, maintained that “mathematical models are not neutral descriptions about an independent reality but that the modelling cycle has devices that are usually concealed to the general public. Since arguments and decisions in society are based on mathematical models, it is important that students can discuss the nature and role of mathematical models” (p. 294). In this respect, Barbosa (2006) emphasised the need for students to engage in reflective discussions regarding the nature of mathematical models.

Although Barbosa (2006) emphasised the importance of reflective discussions, his definition of modelling did not fully and explicitly include aspects of decision-making on controversial issues that are relevant to society or the ethical, moral or economic aspects of these decisions. Therefore, they might be neglected. For example, a classic example of a modelling task without clear links to decision-making and controversial issues relevant to society is the question of how many people are stuck in a 20-km traffic jam (Maass, 2005). The fact that decision-making on societal issues that require the consideration of the ethical, moral or cultural aspects, has not been the focus of attention within the discussion of mathematical modelling, is also mirrored in the classification scheme developed by Maass (2010), which classified modelling tasks according to various aspects (such as steps of the modelling cycle to be carried out, database, connection to reality), but did not mention decision-making.

Numeracy emphasises the aspect of critical citizenship to a greater extent. It refers to the knowledge and competences required to react to private and public life demands and participate in society as responsible and active citizens (Geiger et al., 2015a). There are different definitions and facets of numeracy, such as statistical or financial literacy, workplace numeracy or technical aspects of numeracy (Geiger et al., 2015a). Many of these definitions include a critical component. Being numerate includes the critical judgement of real-world situations and social empowerment, which refers to making conscious decisions and developing arguments that support or challenge the positions created by others (Ernest, 2002; Geiger et al., 2015a). Other definitions emphasise that numeracy is relevant for combatting social injustices and promoting more equitable societies (D’Ambrosio, 2001; Skovsmose, 1994).

**Fig. 2:**
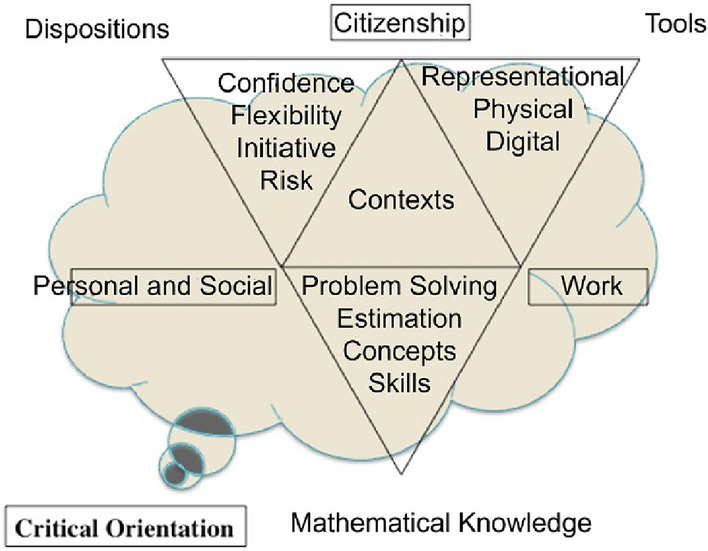
The twenty-first-century numeracy model of Geiger et al., (2015a, 2015b)

Geiger et al. (2015b) argued that the demand for new, societal and sophisticated problem-solving skills in the twenty-first century requires far more than the mastery of basic mathematical skills and procedures. To meet these demands, they described a model of numeracy for the twenty-first century in which mathematics is the foundation for evidence-based decision-making and judgement-forming capabilities that are essential for participatory citizenship (Fig. 2).

The twenty-first-century numeracy model (Geiger et al., 2015b) connects contexts within and beyond school settings, such as personal and social contexts, world of work contexts and contexts relevant to being a responsible citizen, with mathematical knowledge and aptitude for mathematics and tools. All these aspects are essential for being numerate. Additionally, there is an additional aspect that is relevant to being numerate. It emphasises the necessity for people to develop a critical orientation about all the aspects that support them in making evidence-based decisions and judgements (see Fig. 2).

In summary, Geiger et al. (2015b) argued that the demand for societal problem-solving skills in the twenty-first century requires far more than the mastery of basic mathematical skills and procedures. Students need to learn explicitly how to make evidence-based decisions and judgements. Similarly, critical mathematics education (Andersson & Barwell, 2021) integrates the ideas of critical theory. According to these authors, critical mathematics education “is driven by urgent, complex questions; is inter-disciplinary; is politically active and engaged; is democratic; involves critique; and is reflective and self-aware” (p. 3). This approach also highlights global challenges (for example, climate change or the COVID-19 pandemic), which are complex and require urgent mathematical and interdisciplinary solutions. These approaches from numeracy and critical mathematics education are in line with the modelling aim (A3)—Learning to understand their world critically, as discussed in Sect. 2.1.

How can tasks promoting citizenship education be designed? In science, socio-scientific issues (SSIs) involve decision-making based on ethical, economic, political and moral aspects and are core to citizenship education. SSIs, such as the COVID-19 pandemic, are frequently at the frontiers of scientific knowledge, where nobody knows the insights that will be available in four weeks. These SSIs require people to engage in dialogue, discussion and debate (Ratcliff & Grace, 2003) because they are controversial, as revealed by the pandemic situation. Political discussions on the political actions that should be taken (for example, whether a lockdown is required or not) cause controversies among politicians and beyond. This is because political decisions are based, not only on scientific facts, but also on moral, ethical, economic or social issues (Zeidler & Nichols, 2009). Meanwhile, reports about these complex issues in the media do not necessarily state all the information needed, as the communicator’s opinion may bias the presentations (Kwon et al., 2021). People often have to deal with SSIs using incomplete information because of conflicting or insufficient scientific evidence and incomplete reporting. Moreover, these issues involve a cost–benefit analysis in which risk interacts with ethical reasoning (Ratcliff & Grace, 2003). This is clearly observed in the case of the COVID-19 pandemic, where the question of whether to enact a lockdown is connected to a cost–benefit analysis. The COVID-19 pandemic severely affects the physical health of many people. However, is physical health more important than mental health or economic aspects? Such cost–benefit analysis is the basis for decision-making.

### The extended modelling cycle

**Fig. 3:**
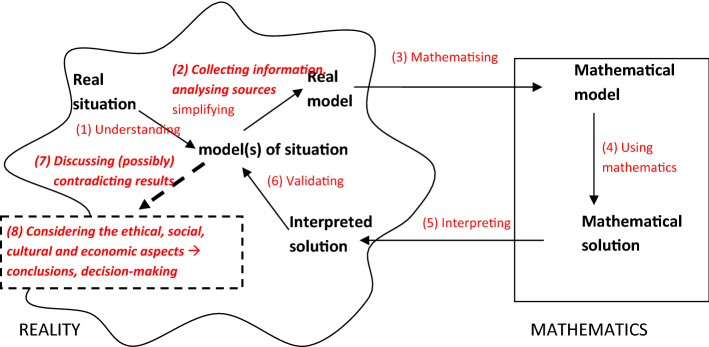
A modelling cycle extended for socio-scientific issues in mathematics education

Maass et al. (2019) shifted the scope of modelling from a focus on purely learning to applying mathematics to real-world contexts, to including capabilities such as evidence-based decision-making, dealing with uncertainty, and fostering critical and scientific mindsets. Following on from the socio-critical perspective (Kaiser & Sriraman, 2006), they suggested extending the modelling cycle to fully consider features of societal issues, such as dealing with controversial information, including ethical, moral, economic or political problems, and requiring people to make decisions. These extensions include collecting information and conducting an analysis of information sources (assessing the quality of media content, social media reports and the credibility of their authors) when going from the model of the situation to the real model. Maass et al. (2019) also suggested including discourse about (possibly) contradicting scientific results and the inclusion of ethical, social and cultural aspects (Zeidler & Nichols, 2009) in decision-making, and drawing conclusions as new steps after validating the modelling results. A possible resulting modelling cycle is shown in Fig. 3. The newly highlighted steps in the modelling cycle (in comparison to the modelling cycle in Fig. 1 of Blum & Leiss, 2007) are highlighted in bold.

Based on our definition of modelling competences as outlined in Sect. 2.2, using this extended modelling cycle also enlarges the number of sub-competences included in modelling competence. The sub-competence (2) now includes collecting information and analysing sources. Additionally, the following two further sub-competences are included: (7) Discussing possibly contradicting results; and (8) drawing conclusions and decision-making by also considering non-mathematical aspects.

These additional sub-competences can be related to the four aims seen for modelling as described in Sect. 2.1. Carrying out these steps can expand students’ learning concerning applying mathematics to their everyday and professional lives (A1), their modelling competences (A2), and their insight into the usefulness of mathematics (A4). Most importantly, working according to this extended modelling cycle can contribute to students’ critical understanding of the world as highlighted by socio-critical modelling (A3). Additionally, tasks requiring students to carry out the extended modelling cycle also target students’ attitudes towards mathematics and their problem-solving competences. Consequently, following the discussion of relevant aims of modelling in Sect. 2.1, this extended modelling cycle is in line with the aims of modelling and is therefore a meaningful extension.

This extended modelling cycle provides a framework for developing and analysing concrete modelling tasks for teaching. Again, we emphasise that the modelling cycle of Blum and Leiss (2007) does not exclude these steps. The modelling cycle, as shown in Fig. 3, only highlights them more explicitly. We use all the steps in Fig. 3 to analyse our examples of tasks. Before we analyse the tasks, we explain their design.

## Design of the generic tasks

The design of the tasks follows the features of design research (van den Akker et al., 2006; Kelly, 2006). They are interventionist and utility-oriented, as they were designed for day-to-day classroom teaching. The examples of tasks were made available for teachers on our website (icse.eu, icse.ph-freiburg.de), teacher platforms, and mailing lists. They are open tasks that students with different performance levels can use. Additionally, they are based on the theory of modelling and are context-oriented in the sense of design research, as the design of the tasks takes into account the context of day-to-day teaching. Finally, they incorporate a (limited) cyclic design, evaluation and revision approach. Due to our aim of supporting students in understanding and critically evaluating the latest developments of the pandemic from the socio-critical modelling perspective (Kaiser & Sriraman, 2006), it seemed essential to us to publish tasks that were concerned with those latest developments. Therefore, the periods for the design, evaluation and revision cycles were limited.

We developed these generic tasks at the International Centre for STEM Education (ICSE) in Freiburg. ICSE is an internationally connected research centre located at the University of Education in Freiburg, Germany. ICSE has the aim of helping improve STEM education across Europe through practice-related research and its transfer into practice. Therefore, the development of teaching tasks that support teachers in students’ citizenship education is essential. Within its daily work, ICSE runs workshops for both students (to raise their interest in STEM education) and teachers (to support them in their professional development), and develops related materials (such as the tasks related to the COVID-19 pandemic).

A mathematics teacher from ICSE designed examples of tasks for lower and higher schools. Afterwards, another teacher screened them for important characteristics, including relevance, effectiveness (for learning about the COVID-19 pandemic and how to model) and practicability (task usability in lower secondary classes). Subsequently, they were discussed with an expert on modelling (expert appraisal, Nieveen, 2007) and optimised accordingly.

## Didactical analysis of three examples of tasks

In the following sections, we show how the context of the pandemic can be used to develop tasks that connect modelling to citizenship education. We provide and discuss three generic tasks that focus on different aspects of the connection between modelling and citizenship education. Subsequently, we analyse them didactically based on the extended modelling cycle as described in Sect. 2.3.

### Task 1: a task with pre-defined models.

*The task and a possible solution of students*

This task deals with virus variations and how they affect possible measures.
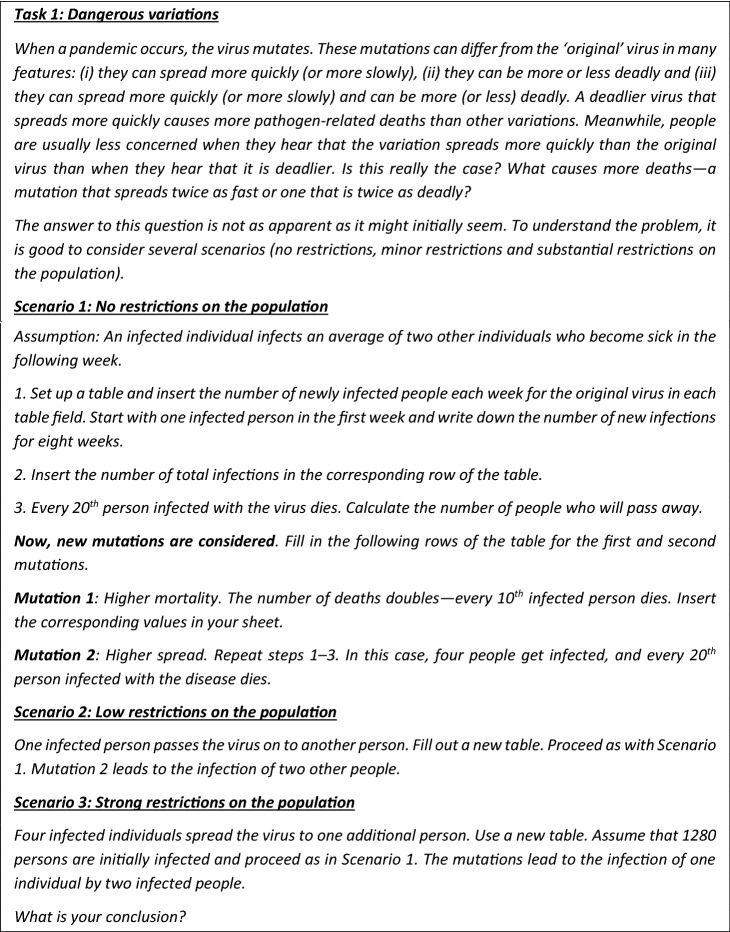


Firstly, we reflect on how students can solve the task.

**Fig. 4:**
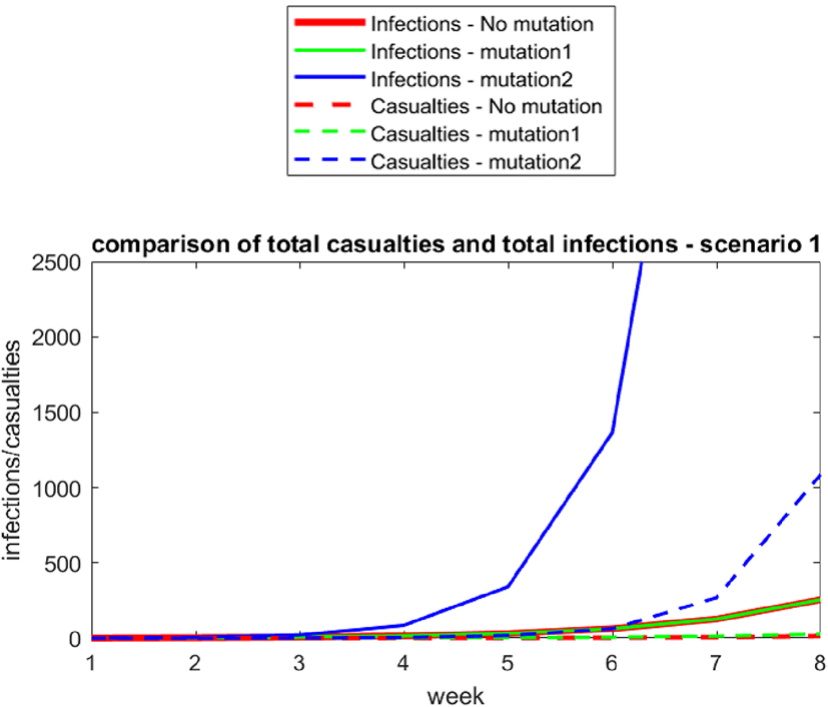
Scenario 1—Infections and casualties for the original virus, mutations 1 and 2

**Fig. 5:**
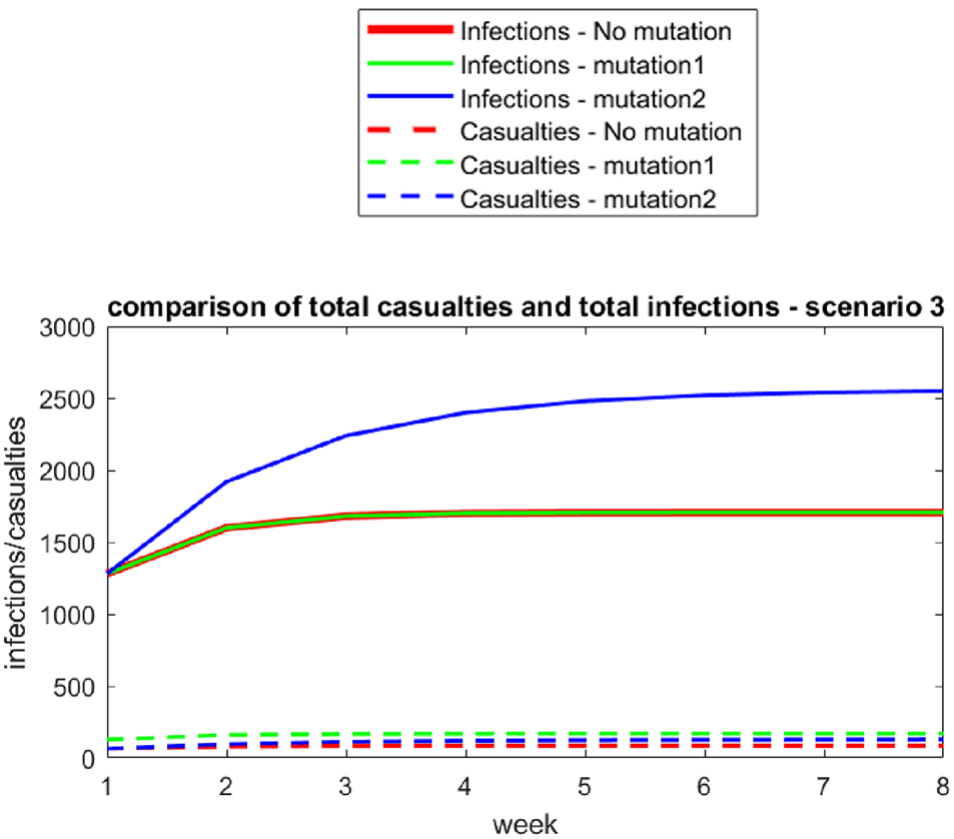
Scenario 3—Infections and casualties for the original virus under strong restrictions on the population

In this task, the mathematical models are already given. So, students can enter the extended modelling cycle by carrying out step 4 (using mathematics). (Students must also understand the situation, in step 1, and they are required to transfer the given mathematical model into tables). For finding a mathematical solution, young students would probably set up tables, as required in the task, and then insert every single number. Older students may adopt formulas after starting to fill in the table because this procedure is much quicker. They might end up with graphs, as shown in Figs. 4 and 5, as mathematical solutions.

When interpreting the mathematical solutions (the graphs) (step 5), students might notice that the number of cases increases significantly, revealing the enormous difference between the effects of the original virus and mutation 2. In the beginning, with one person infected, 13 (255/20 = 12.75 ≈ 13) casualties are expected with the original virus. With mutation 1, the number of deaths increases to 26, and with mutation 2, the number of deaths is 21,745/20 = 1087.24 ≈ 1087. Consequently, mutation 2 has a much more devastating impact, which might be surprising at first, as one might think that a deadlier mutation is more dangerous than a mutation that spreads more quickly.

In Scenario 2, the situation looks very different (for reasons of space, we do not show the graphs here). The number of cases is 8 for both the original virus and mutation 1 but 255 for mutation 2. The number of casualties is below 1 for the original virus, 1 for mutation 1 and 255/20 = 12.75 ≈ 13 for mutation 2.

In Scenario 3, students may notice the decreasing impact of the strong restrictions. The number of deaths with the original virus is 1706.57/20 ≈ 85.33 ≈ 85. With mutation 1, it is approximately 170. With mutation 2, approximately 2950/20 = 147.5 ≈ 148 infected individuals would pass away.

When validating the model (step 6) students would need to check whether these results are realistic. As the results could be surprising for many students, this task points out the necessity of validating the results, which is a step of the modelling cycle that students often neglect (Maass, 2004). Students could also compare the results with real data from the pandemic. New mutations might not spread twice as fast, and restrictions might not lead to the effects as assumed here and other assumptions might be used. Nevertheless, the modelling shows how different mutations impact the situation differently.

There are no contradictory results here that need to be discussed (step 7 of the extended modelling cycle). What conclusions (part of step 8) can be drawn from these data? The results show that answering the question regarding which mutation leads to pathogen-related deaths is difficult. In the first two scenarios, the mutation with a higher spread caused the most deaths. Under the strict lockdown of Scenario 3 (the lockdowns in Germany and Austria were by far not as strict), the mutation with the higher death rate caused more pathogen-related deaths. Combining the results from all the models, it shows that mutation 1 is more dangerous for those who know that they are infected, whereas mutation 2 is a more significant threat for those who are not yet infected, as well as the society as a whole.

*Didactical analysis of the task*

As has been shown in the possible solution above, all steps of the extended modelling cycle from step (4) to step (8) must be carried out. However, students do not explicitly learn to make autonomous and well-founded decisions based on the results of the modelling cycles. As students are confronted with different scenarios, they can compare different models. Hence, understanding the models’ nature and consequences is a preparatory step for decision-making.

As not all steps of the extended modelling cycle have to be carried out (there may be no need to set up a real or a mathematical model), the task is an atomistic modelling task. Different models or scenarios are given, and students must work with them mathematically. Students might start with the mathematical model and be required to find a mathematical solution, interpret the solution (the graphs) validate them and draw conclusions. Still, as has been said above, the task has a high didactic potential in serving the overall goal of developing students’ modelling competences. It offers students insight into how different assumptions can lead to different results. The task already provides all the data needed for the calculation; thus, finding and analysing additional sources is unnecessary (step 2 of the extended modelling cycle). As Mischo and Maass (2013) showed in their empirical study, such atomistic tasks can contribute to developing students’ modelling competences if holistic modelling tasks are also dealt with. Our second generic task is such a holistic task.

### Task 2: a holistic modelling task

*The task and a possible solution of students*

 Task 2 focuses on carrying out a whole modelling cycle and therefore is a holistic task.
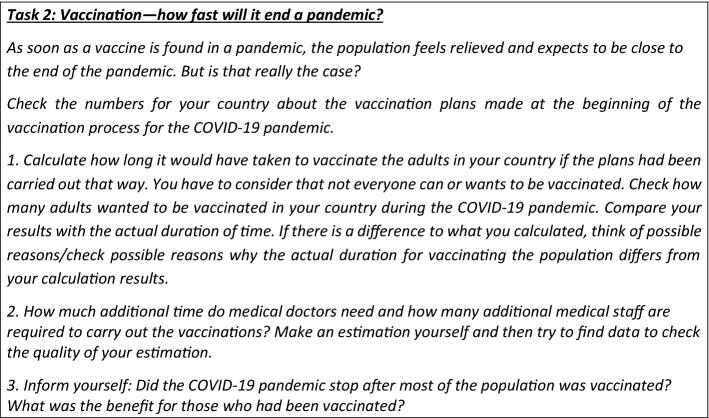


Before we analyse the task didactically, we reflect on how students could solve the task.

First, students have to understand the real situation (step 1 of the extended modelling cycle), including the following aspects: (a) When the vaccine was newly developed and accredited, it was impossible to obtain sufficient vaccines instantly. (b) According to daily news and media, some people felt insecure about the vaccine and hesitated to get vaccinated. All these aspects make predictions (of when the pandemic will end) difficult. After modelling the situation, these aspects must be revisited.

Because the task demands it, students might have to collect information by searching online journals or asking family and friends how many people live in the country, how many people want to get vaccinated, how many people will be vaccinated every day and how many doctors are needed (consequently, step 2 of the extended modelling cycle). The answers to these questions might differ depending on the sources students use, and these differences and their impact on the outcome of the modelling could be discussed at this stage. Furthermore, students may learn to be careful about their information sources, as not all resources are reliable with such an emotion-stirring topic as the COVID-19 pandemic (thus analysing sources as required in step 2).

We now show two examples of calculations based on different sources that lead to very different results. We show how—based on the different information sources—different mathematic models (step 3) are set up and lead to different solutions (step 4) and thus to different interpretations of the results (step 5).

In the first source,^1^ the central vaccination centre of Freiburg is one of the eight vaccination centres in Baden-Württemberg, and 1500 people would be vaccinated daily in each centre. The assumption was that approximately 65% of the population could and wanted to be vaccinated. Based on these numbers and considering that Baden-Württemberg has approximately 11,000,000 inhabitants, one can calculate that it would take about 0.65 × 11,000,000/(8 × 1500) ≈ 600 days to vaccinate enough people once.

The second source^2^ states that nine central vaccination centres and about 50 regional vaccination centres will be built. Approximately 800 people are supposed to be vaccinated daily in each centre. This means that it takes 0.65 × 11,000,000/(59 × 800) ≈ 150 days to vaccinate enough people once.

In reality, it took about eight months $(\approx$ 240 days) to vaccinate approximately 65% of the people in Baden-Württemberg.^3^ When answering the second question of the task, the same problem can occur with different sources.

When validating the result (step 6 of the extended modelling cycle), students might need to reflect on whether they considered all the contextual conditions needed (such as different vaccination numbers on weekends compared to weekdays or an increasing number of available and permitted vaccinations as time goes by). The task also allows a ‘reality check’, which is rarely the case with modelling tasks: students can compare their results to the real-world situation (for example, the time it took to vaccinate everyone in the population who wanted to get vaccinated).

*Didactical analysis of the task*

By being asked to compare their modelling results with real data, students have an opportunity to engage in reflective discussions, which is emphasised by Barbosa (2006). This reflection, in turn, prepares students for decision-making based on modelling. However, a decision based on scientific arguments (including ethical, moral, economic or political aspects; see step 8 of the extended modelling process) is not required (Fig. 3). A discussion of contradicting results (step 7) might be only necessary if students use very different resources.

This task is a typical holistic modelling task. Students may go through the whole modelling cycle proposed by Blum and Leiss (2007). Consequently, they can learn to perform a modelling cycle in a complex situation. Unlike Task 1, most students might probably perform calculations with only one set of assumptions, thereby obtaining only one solution. Thus, although students need to carry out the whole modelling cycle in this task, seeing how different solutions develop using different assumptions is missing. However, the impact of different assumptions is reflected by asking students to compare their results with the time needed in the real world. Furthermore, when comparing solutions from all students in the class, the impact of different assumptions may become evident; however, students might not experience this anomaly within their own calculations. Thus, using both generic tasks in class is of added value.

Unlike Task 1 not all data are given here, therefore step 2 of the extended modelling cycle (collecting information) is relevant here. Hence, students must search for data on the internet and critically analyse the sources.

### Task 3: modelling, including decision-making

*The task and a possible solution of students*

The next task requires carrying out a modelling cycle and explicitly includes decision-making.
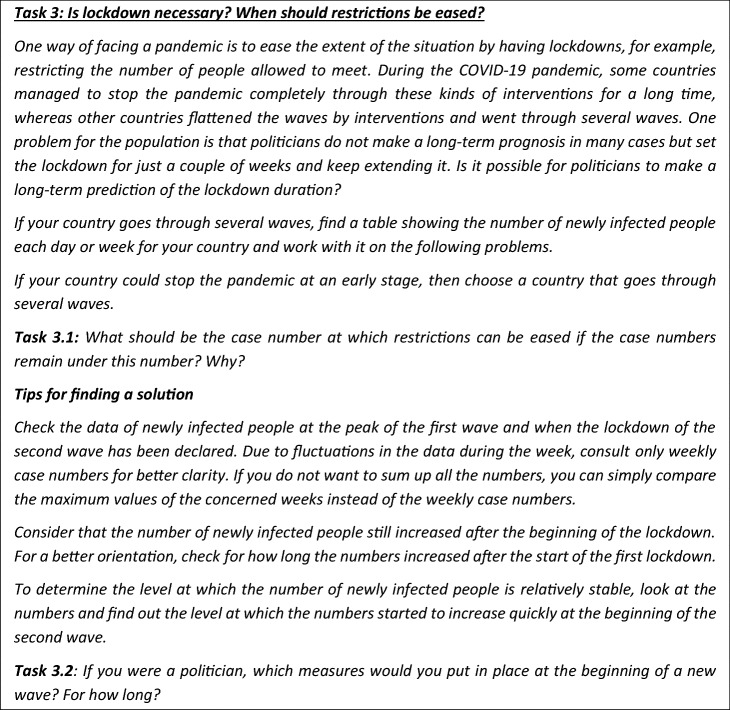


In the following paragraphs, we present a possible solution.

**Fig. 6:**
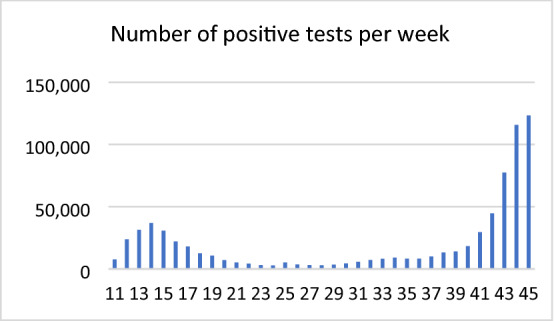
Number of positive tests per week (starting from week 11) in Germany

Students first must understand the situation (step 1). Then students might have to search for information (step 2 of the extended modelling cycle)—in this case, the number of infected people. Here, they may have to decide whether to consider daily numbers or the number of infected people per week. This possible solution takes the number of infected people per week, as daily numbers can be subject to strong fluctuations. Figure 6 shows the number of positive tests per week from which the numbers are taken.^4^ To use the information given in the graph, students need to read the graph and interpret it.

How could students model this situation to decide when politicians should enact restrictions and what kinds of restrictions should be enacted?

The following reflections might guide the setting up of the real model (step 3) and the mathematical model (step 4). From the graph, approximately 20,000 infected people (or 15,000 or 25,000) may be regarded as a turning point. Below 20,000 the numbers seem to increase relatively slowly, and from week 40–41 (increasing from 18,920 to 29,567), the slope appears to be much steeper than before. A closer look at the data reveals that the steep rise has two reasons. One reason is that the virus spread is exponential; consequently, the slope increases exponentially. The other reason is that the basis of the exponential growth (the reproduction index) increases over time. The reproduction index is between 1 and 1.3 until week 40. From week 41, it is above 1.5 and gradually approaches 2.

Suppose, for example, that one decides to model this fuzzy situation with an exponential function based on a reproduction index of 1.7. This would allow one to predict the number of infected people more or less accurately. However, neither looking at the exponential function graph nor the increasing reproduction index enables one to deliver a precise measurement of the point at which restrictions should be taken and what these measures should be. Based on the graph, it is only clear that the longer one waits, the longer it will take to bring the numbers down again to an acceptable level. Thus, this model will not lead to a decision concerning what political measures should be taken. Therefore, other measures also need to be considered in setting up a model (step 3 and step 4 of the extended modelling cycle). For example, one can consider the number of intensive care units (ICUs). In Germany, there are about 25,000 ICUs.

Students might use the following assumptions for setting up a model (steps 3 and 4) and interpreting it (step 5): COVID-19 patients can occupy 20% of the ICUs without neglecting other patients (consequently 5,000 ICUs are available in Germany), 10% of COVID-19 patients go to hospital and 20% of them need an ICU. Thus, if there are 20,000 infected people, 280 ICUs are required. However, if there are 80,000 infected people, 1120 ICUs are needed. Thus, could 80,000 be a number at which restrictions are required? Students could validate the results as follows (step 6): If one assumes a reproduction index of 1.7, and after two weeks an index of 1.5 and later 1.3, one sees that the ICU capacities are exceeded. Thus, modelling the situation in more detail offers more orientation but still delivers no precise measurement. Thus, a reflective discussion on the possibilities and limitations of mathematics and mathematical modelling is needed (Barbosa, 2006). Students can learn that mathematics does not provide an unambiguous solution to questions.

If one takes 40,000 infections as a milestone for restrictions and if the other assumptions are correct (which the student needs to review in the validation process, step 6), the risk of exceeding the available ICU capacities is smaller. However, the question of which measures precisely need to be taken is still not answered and requires further modelling. Also, there will be controversial results based on the different assumptions made, which need to be discussed (step 7).

Then, step 8 of the extended modelling cycle comes in explicitly. The measures taken at a specific time depend mainly on non-mathematical aspects. From a political point of view, one may hesitate to decide on a lockdown so early because, at this point, the population may not see the ‘danger’. Indeed, in Germany, measures were taken in the second wave at 80,000 new cases per week. Considering the measures from an economic point of view, it is best to close schools, universities and especially nursing homes, because these are places where many people gather. Nevertheless, it causes no immediate economic damage—at least in the short term. This, however, neglects the social aspects and mental health of children and older adults. Considering this perspective, universities and nursing homes must be kept open because these measures isolate the most vulnerable groups. Instead, a politician might decide that adults should work from home to minimise contact in transportation facilities and workplaces. Furthermore, the politician might decide to close venues of social activities, such as amusement parks, discos and pubs, as they fuel a spread while not being crucial for mental health and the economy. This decision heavily depends on non-mathematical aspects, such as economic aspects, medical and ethical aspects.

*Didactical analysis of the task*

This task explicitly includes decision-making based on modelling, and thus step 8 of the extended modelling cycle. In this task, it becomes evident that decision-making also includes several non-mathematical aspects. Such tasks help students dealing not only with the pandemic but also with other societal challenges and are therefore of utmost societal relevance. For example, we could also develop tasks to cope with climate change in this way. In this task, students have the opportunity to learn about the relationship between mathematical modelling and decision-making. Thus, they can learn *about* mathematics, which is education in the sense of responsible citizenship and prepares them for an enlightened and critical worldview. Consequently, this task, more than the other two, highlights an aspect emphasised in the socio-critical modelling perspective and in the discussion of numeracy (Geiger et al., 2015a). The extended modelling cycle suggested by Maass et al. (2019) also stresses this aspect. This task is holistic (Blomhoej & Jensen, 2003), as it requires carrying out all steps illustrated in Fig. 3.

## Summary and discussion

To enable individuals to react as responsible citizens in situations such as the COVID-19 pandemic, they need to have a a profound understanding of mathematical modelling cycles. They also need to be equipped to make autonomous and well-grounded decisions based on the results of modelling cycles, bearing in mind that these decisions are also based on non-mathematical aspects (e.g., ethical, moral or cultural facets). This is how mathematical modelling is linked to citizenship education. Since these issues are not always the focus of mathematical modelling cycles or their respective theoretical classifications (Maass, 2010), we discussed how the theoretical framework of mathematical modelling could be complemented explicitly to link to citizenship education. To realise this connection in theory and show how this connection can be used in teaching practice, we developed three different generic modelling tasks and analysed them didactically.

Developing and discussing these tasks also highlights some limitations. Since the COVID-19 pandemic is rather dynamic, we had limited time in designing and evaluating the tasks. Due to these time restrictions and school lockdowns, there was no possibility of a formal try-out and pilot phase in class before publishing the tasks, as suggested in design research. This is a significant limitation since additional perspectives (for example, of teachers, pupils and subject and didactics experts) would offer further insights into the strengths and weaknesses of the tasks.

Concerning the topic of the tasks, we understand that the context of the COVID-19 pandemic can also be a sensitive topic. A student may have lost relatives because of the pandemic or the family may be experiencing financial difficulties because family members lost their jobs. Here, the teachers must balance benefits and possible problems and, in any case, deal with such tasks in a sensitive way.

The discussion of our three tasks shows that it is not only possible to link mathematical modelling explicitly with citizenship education in theory but also possible to develop tasks that transfer these links into practice. To implement such tasks, related lessons in professional development courses should also be designed. Maass et al. (2019) discussed the content of such a course and Maass et al. (2021) analysed its impact on teachers. They found that teachers’ self-efficacy beliefs and their learning-related beliefs about using such tasks for citizenship education, as well as their own teaching practice, changed significantly after participating in the professional development course.

To widen the scope, such tasks can also deal with different societal challenges, such as climate change (Barwell & Hauge, 2021; Steffensen et al., 2021). Similarly, other mathematical content and concepts (beyond modelling) are also of interest and could be highly valuable for discussing these issues. For example, statistical literacy, functional thinking and epistemology are imperative for understanding global challenges. Thus, the focus of this paper on modelling cycles is not the only possible way to look critically at tasks. However, particularly regarding the actual pandemic situation, it is a viable and valuable strand to further develop knowledge and practices in mathematics education.

## Footnotes

1 https://www.badische-zeitung.de/impfzentrum-auf-der-messe-freiburg-soll-mitte-dezember-einsatzbereit-sein--198227138.html.

2 https://sozialministerium.baden-wuerttemberg.de/de/service/presse/pressemitteilung/pid/standorte-fuer-impfzentren-im-land-stehen-fest/.

3 https://www.rki.de/DE/Content/InfAZ/N/Neuartiges_Coronavirus/Daten/Impfquoten-Tab.html.

4 https://www.rki.de/DE/Content/InfAZ/N/Neuartiges_Coronavirus/Fallzahlen.html.
